# Modifications of Human Subcutaneous ADMSC after PPAR*γ* Activation and Cold Exposition

**DOI:** 10.1155/2015/196348

**Published:** 2015-08-03

**Authors:** Diana Vargas, Wendy Rosales, Fernando Lizcano

**Affiliations:** Centro de Investigación Biomédica, CIBUS, Universidad de La Sabana, Km 7, Autopista Norte de Bogotá, 140013 Chía, Colombia

## Abstract

Mesenchymal stem cells are a diverse population of cells with a wide range of potential therapeutic applications. In particular, cells from adipose tissue have the distinction of being easily accessible and contain a lot of stem cells. ADMSCs can be induced to mature adipocyte and activate the energy expenditure upon treatment with total PPAR*γ* agonists. Additionally these cells may respond to cold by activating the thermogenic program. In the present study, we determined the effect of partial agonism of PPAR*γ* and temperature reduction on phenotype and metabolic activity of ADMSCs from human adipose subcutaneous tissue. We found that adipocytes differentiated with total and partial agonists of PPAR*γ* and exposed to 31°C are able to respond to cold significantly increasing the expression of thermogenic proteins such as UCP1, PGC1*α*, and CITED1, a marker of beige phenotype. Additionally, we found that adipocyte cells subjected to cold had a reduction in triglycerides and increased adiponectin levels. These data confirm the promising role of ADMSCs as a treatment for metabolic disorders since it is possible to induce them to mature adipocytes and modulate their phenotype toward a cell with high-energy expenditure and metabolic beneficial effect.

## 1. Introduction

Stem cells derived from adult tissue have acquired an important role in regenerative medicine as well as a model to determine the origin of chronic nontransmissible diseases [1]. Mesenchymal cells derived from adipose tissue have a special connotation because they are easily obtained and have a great capability to transform [2, 3]. Due to this competence of autorenewal and differentiation in cellular types such as osteoblasts, myocytes, chondrocytes, and adipocytes, ADMSCs (adipose-derived mesenchymal stem cells) are being introduced in a number of therapies of tissue regeneration [4, 5]. A possible therapeutic application of ADMSCs is a phenotypic modulation to treat obesity, a disease considered pandemic with multiple metabolic and cardiovascular repercussions [6]. Since therapeutical resources to treat obesity are scarce, the use of ADMSCs as a model of obesity treatment has become a plausible alternative [7–9]. The capacity of ADMSCs to acquire a determined phenotype depends on specific transcription factors. Activation of PPAR*γ*, a nuclear receptor, which may be activated by specific ligands, can differentiate ADMSCs towards adipocytes. We have previously observed that both total PPAR*γ* agonism with Rosiglitazone and partial agonism with Telmisartan can differentiate unipotent mice cells 3T3-L1 towards adipocytes [10]. However, little is known about partial activation of PPAR*γ* in adipocytes differentiation of ADMSCs [11]. It has recently been observed that it is possible to obtain more active calorigenic adipose cells from human adults, a fact only observed in newborns and inferior mammals that have brown adipocytes [12]. In this contest the adipose cells in adults may be white, brown, and beige. The origin of brown adipose cells is closer with muscle cells, whereas beige adipocytes maybe in part arise from white adipose cells or have their own mesenchymal origin [12–14]. In fact, continuous stimulation of PPAR*γ* receptors is capable of activating the beige phenotype from ADMSCs obtained from human tissue. Even in mice, total agonist of PPAR*γ* is necessary to induce beige phenotype [15, 16]. Additionally, adipose cells can be more active in the production of heat after temperature reduction. Exposure to cold activates the sympathetic nervous system for the liberation of norepinephrine, which acts on *β*-adrenergic receptors, and triggers a signaling pathway that activates the transcription of genes involved in thermogenesis and energy expenditure [17, 18]. Nevertheless, chronic cold exposition may induce thermogenesis and browning of WAT fat, through alternate pathway that may include activation of macrophages and activation of adenosine A2a receptors [19, 20]. From the metabolic point of view, cold generates weight reduction and improves the metabolism of triglycerides by increasing lipids uptake in the brown adipocyte tissue and modulates the expression of some adipocytokines as adiponectin [21].

We use ADMSCs from subcutaneous human tissue to investigate whether pharmacological stimulation with total and partial agonist of PPAR*γ* can activate traits of beige adipocytes as well as to respond to cold activating the thermogenic program. Additionally, we studied the metabolic effect on the levels of triglycerides and the activation of adiponectin under the circumstances described above.

## 2. Material and Methods

Samples of subcutaneous fat were obtained from 5 female patients, with average age of 31 ± 4 (20 to 40) years, who underwent abdominoplasty. Patients presented a BMI (body mass index) between 23 and 25 kg/m^2^. Additionally, donors were not under any treatment with drugs 3 months prior to taking the sample, hence not presenting with any kind of disease.

Lipid profile, carbohydrates metabolism, and thyroid function were within normal levels. Patients received detailed information regarding the objective of the study and signed the informed consent. The project was approved by the ethics committee from Universidad de La Sabana.

### 2.1. Cell Cultures

ADMSCs were isolated from 30 g of subcutaneous abdominal adipose tissue collected during surgery. Fat samples were washed using PBS and all fibrous material and visible blood vessels were removed. After the samples were digested with 250 U/mL collagenase type I, 20 mg/mL BSA, and 60 *μ*g/mL gentamicin in PBS for 90 minutes at 37°C in agitation. After the digestion the samples were centrifuged at 200 g for 10 minutes and pellet was suspended in a solution of erythrocytes lysis composed of 154 mm ammonium chloride (NH_4_Cl), 5.7 mm monobasic potassium phosphate (K_2_HPO_4_), and 0.1 mM EDTA pH 7.3 for 10 min. This mixture was filtered on a nylon mesh of 150 *μ*m pore, followed by centrifugation at 200 g for 10 min. Then the cell pellet was suspended in growth medium consisting of DMEM/F12 plus 10% fetal bovine serum and gentamicin 50 *μ*g/mL at a density of 10,000 cells/cm^2^. After 24 hours the cells were washed and induced to proliferation in medium PM4 (DMEM/F12, 2.5% fetal bovine serum, 1 ng/mL of basic fibroblast growth factor, 10 ng/mL epidermal growth factor, and insulin 8.7 *μ*M) up to 100% confluence and subjected to differentiation into mature adipocytes. Cells were identified with the marker CD34 by flow cytometry.

### 2.2. Differentiation and Quantification of Triglyceride

Human mesenchymal stem cells were differentiated into adipocytes using a mixture of 66 nM insulin, 1 nm triiodo-L-thyronine, 10 *μ*g/mL transferrin, 0.5 mm isobutylmethylxanthine, dexamethasone 100 nM, 1 *μ*M of Rosiglitazone, or 50 *μ*M of Telmisartan as PPAR*γ* agonists in DMEM/F12 for 72 hours. Subsequently, the medium was changed to basal preadipocytes medium containing equal concentrations of insulin, triiodo-L-thyronine, and transferrin alone for 10 days, changing the medium every 3 days. Adipocyte differentiation was observed by staining with Oil Red O, previously fixing mature cells with 10% formaldehyde in PBS for 15 m at 37°C; the solution of ORO in isopropanol was then added for 2 hours at room temperature. It was subsequently removed and washed with water to remove residual dye. To quantify triglyceride, 1 mL of isopropanol was added for 5 min, to distain the fat deposits. Absorbance was measured at 510 nm wavelength and the relative value of triglycerides was determined.

### 2.3. Cold Induction

ADMSCs were cultured in 6-well boxes, induced to adipogenic differentiation, and maintained for 10 days at 37°C in basal differentiation medium. 18 hours before cold induction, mature adipocytes were washed with PBS and the basal differentiation medium was changed. Subsequently, cells were subjected to 31°C during 4 hours in an incubator with 5% CO_2_. After this time, the adipocytes were stained with ORO for quantification of triglyceride and other lysates for total protein extraction. Respective controls were cells under similar conditions of differentiation maintained at 37°C.

### 2.4. Western Blot Analysis

Total protein differentiated adipocytes from subcutaneous fat were obtained by using RIPA buffer (Abcam, Cambridge, MA, ab156034) and 1 *μ*g Protease Inhibitor (Roche Diagnostic, Mannheim, Germany). They were then quantified using the Bradford method to work with a concentration of 50 *μ*g of protein. It was followed by denaturation at 95°C that was conducted to subject the extracts to electrophoresis in polyacrylamide gel. Subsequently the product of electrophoresis was transferred to PVDF membrane pretreated with 100% methanol for 2 min.

Blocking of the membrane was performed with PBS-T (1X PBS and Tween 20 0.1%) and nonfat milk at 5%. Then it was incubated with the respective antibodies to detect thermogenic proteins: rabbit anti-PGC-1*α* (peroxisome proliferator-activated receptor gamma coactivator 1-alpha), 1 : 1000 dilution (Abcam, Cambridge, MA, ab54481), rabbit anti-UCP-1 (uncoupling protein 1), dilution 1 : 1000 (Abcam, Cambridge, MA, ab155117), and mouse anti-CITED1 (Cbp/p300-interacting transactivator with Glu/Asp-rich carboxy-terminal domain) (Abcam, Cambridge, MA, ab87978). Secondary antibodies against rabbit IgG-HRP for UCP1, PGC-1*α* and a dilution of 1 : 2000 and 1 : 3000, respectively (Abcam, Cambridge, MA, ab6721). Furthermore, mouse IgG-HRP (Abcam, Cambridge, MA, ab6728) was used for CITED1 at a dilution of 1 : 2000. In addition, the expression of adipocytokines as FABP4 (fatty acid binding protein 4) was detected using a rabbit antibody anti-FABP4 (Abcam, Cambridge, MA, ab92501) at a dilution of 1 : 2000 and adiponectin (Abcam, Cambridge, MA, ab75989) at a dilution of 1 : 3000, and as secondary antibody rabbit IgG-HRP was used at 1 : 5000. Detection was performed by chemiluminescence according to the instructions of Luminata Crescendo Kit (EMD Millipore, Darmstadt, Germany). Images were captured and analyzed using myECL Imager (Thermo-Scientific, Waltham, MA). Quantitative analysis was performed by densitometry using my Image Analysis program in 3 independent experiments. Results were subjected to the test of variance analysis (ANOVA) and differences were considered statistically significant when the value of the mean with standard error was *P* < 0.05.

### 2.5. Treatment of Adipose Cell with IL4

In order to know the effect of IL4 on thermogenesis, ADMSCs obtained from 3 female patients were grown in 6-well boxes and differentiated as described above. Mature adipocytes were washed with PBS and treated with IL4 10 ng/mL (PeproTech, Rocky Hill, NJ) in 0.1% BSA. Cells were immediately subjected to 31°C or 37°C for 4 hours. The respective controls were cells treated only with BSA. Total proteins were obtained to perform western blot analysis and were incubated with the respective antibodies for adiponectin and PGC-1*α*. The experiments were performed in triplicate. Quantitative analysis was performed as described above.

## 3. Results and Discussion

Stem cells derived from adipose tissue (ADMSCs) are a useful tool for the study of different types of diseases. We obtained ADMSCs from subcutaneous adipose tissue of healthy women as a model to assess the effect of adipocyte differentiation with different PPAR*γ* agonists and also to observe the influence of temperature on the development of thermogenic and metabolic markers. After establishing the conditions for differentiation and culture we obtained ADMSCs that were identified by flow cytometry using the CD34 marker [22, 23].

**Figure 1:**
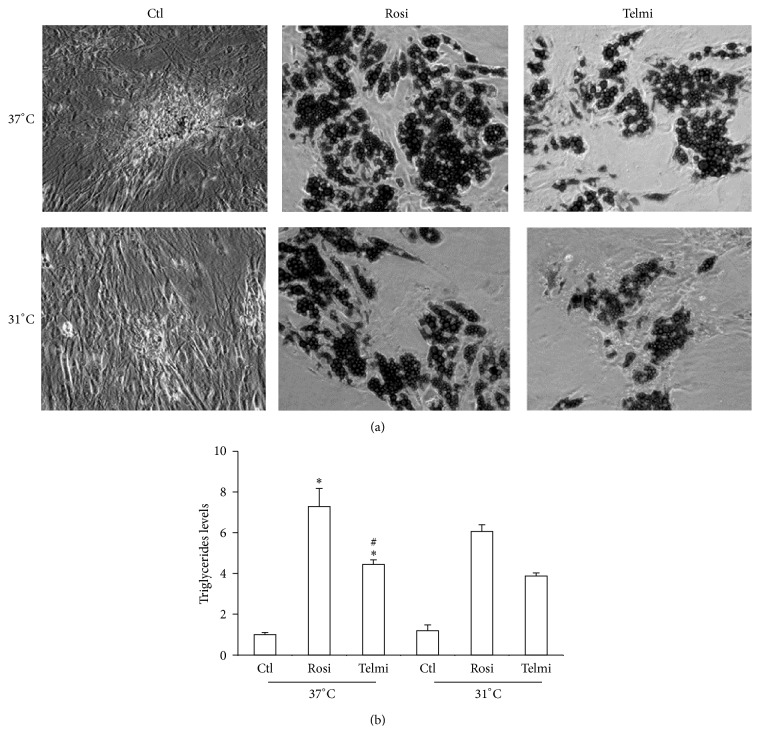
Effect of cold on triglyceride levels in differentiated human adipocytes. (a) ADMSCs were obtained from human subcutaneous adipocytes differentiated and induced for 10 days with 1 *μ*M of full agonist (Rosiglitazone) and 50 *μ*M partial agonist (Telmisartan). Cells were maintained at 37°C or exposed to 31°C for 4 hours and after, staining was performed with Oil Red O. (b) Quantification of triglyceride levels is expressed as relative values. Statistical analysis was performed using ANOVA test. Data are expressed as mean ± SD of 3 independent experiments performed in triplicate. Differences were considered statistically significant at *P* < 0.05. Rosi (Rosiglitazone), Telmi (Telmisartan). ∗Differences between triglyceride levels of treatments with PPAR agonist in relation to control (induction of differentiation with basal medium). # indicates differences found between treatments.

**Figure 2:**
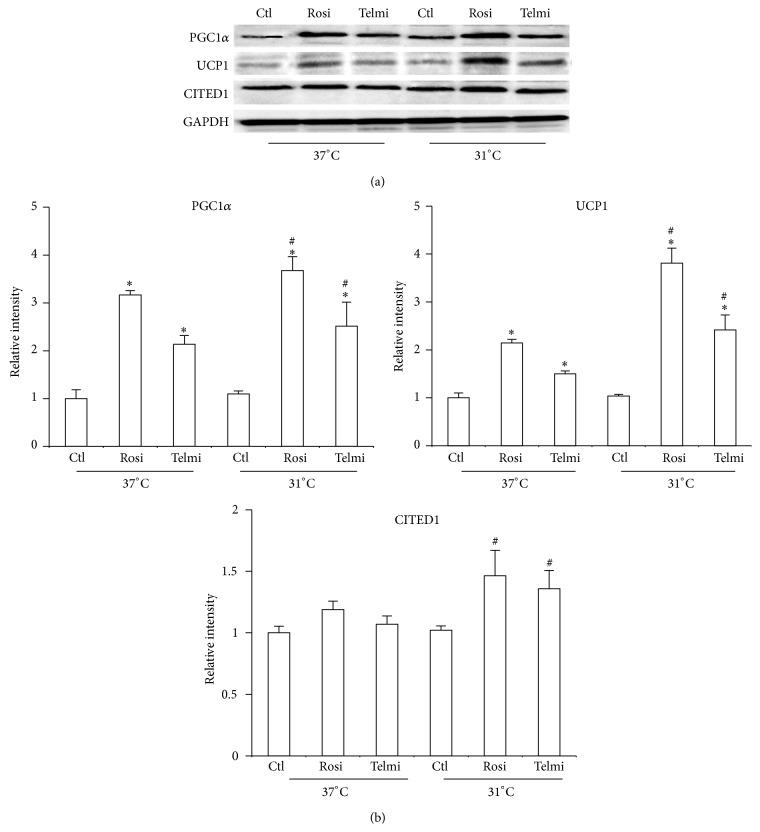
Effect of cold and full and partial agonists in the adipocyte phenotype. (a) Differentiated adipocytes were obtained and treated as described in Figure 1; total protein was isolated for detecting expression of PGC1*α*, UCP1, and CITED1 using western blot. (b) Relative intensity levels were determined by densitometry of bands. Analyses were performed using ANOVA test. Data are expressed as mean ± SD and differences were considered *P* < 0.05. ∗Differences between total and partial agonist in relation to control. # indicates meaningful differences observed after 4 hours of treatment with agonists after exposure to cold in comparison with adipocytes treated in equal conditions at 37°C. Data were normalized with GAPDH.

The effect of PPAR*γ* agonist was initially studied, whether partial or total, to induce differentiation of ADMSCs into adipocytes. After 10 days of differentiation it was observed that cells were capable of storing triglycerides using both agonists (Figure 1). This observation makes the important role of this nuclear receptor in the process of differentiation of the adipose cells evident. In the present study both total and partial PPAR*γ* agonists induced triglyceride accumulation and differentiation of ADMSCs to mature adipose cells. We have considered the evaluation of a partial agonist of PPAR*γ* because it has been shown that excessive fat accumulation and retention of liquids are some of the undesirable effects of total PPAR*γ* agonists. It is for this reason that selective agonists may have a better therapeutic efficacy [24, 25]. When cells were subjected to a temperature drop, in both cases a reduction in the accumulation of triglycerides was observed (Figures 1(a) and 1(b)). Previous studies in animal models have shown that cold can possibly have a lipolytic effect on fat cells, increasing transporters of fatty acid and specific enzymes such as lipase lipoprotein [26]. It was recently observed that the reduction in temperature can steadily increase expression of higher heat production genes [17]. An important event observed in this study is the increase in markers of energy expenditure triggered by the two types of agonists. Even though it was previously observed that Rosiglitazone may increase PGC-1*α* and UCP1 levels [27], this is the first evidence of the effect of a PPAR*γ* partial modulator in the activation of these markers (Figure 2). It was additionally observed that temperature reduction may increase the production of markers of energy expenditure, more evident in the case of Rosiglitazone as compared to Telmisartan.

The reduction in temperature has been shown to have a physiological impact on the activation of thermogenic markers of adipose cells. In some animal studies it was found that cold can increase thermogenesis markers and possibly adipose cells have a functional change [28]. In this work, human adipocyte cells underwent a temperature reduction from 37°C to 31°C, keeping it during 4 hours. After being at 31°C during this period, the cells increased the expression of thermogenesis markers as UCP1 and PGC-1*α* (Figure 2). The activity of the sympathetic nervous system that stimulates *β*-adrenergic receptors may be important for thermogenesis activation in response to cold. Our data show that both partial and total PPAR*γ* agonist may modify the expression of protein involved in energy expenditure and strikingly the cells can respond to cold increasing the thermogenesis [17]. Hence it is possible that, besides sympathetic nervous system mediation, some additional factors may be important for the phenotypic induced by cold. It has been recently demonstrated that activation of adenosine receptor or IL4 production is probable factors that mediate this functional change at low temperatures. However, the mechanism by which cold can modify the activity of adipocytes is still to be elucidated [20].

**Figure 3:**
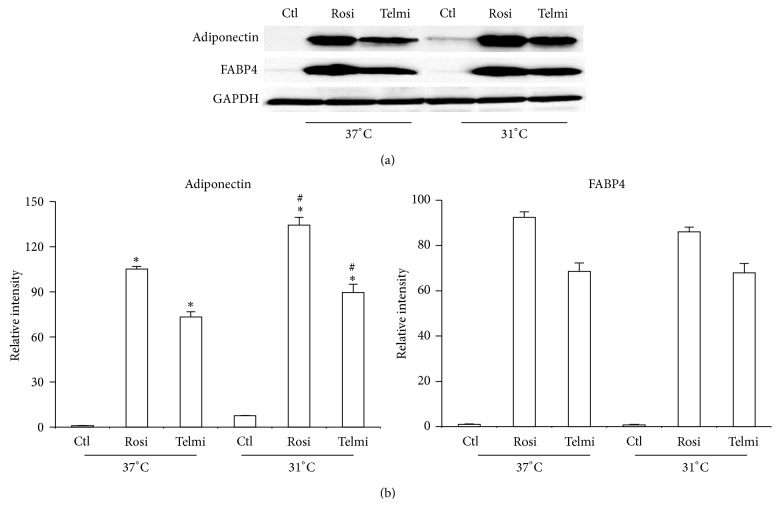
Effect of cold in the levels of adipocytokines. (a) The differentiated adipocytes were obtained and treated as described in Figure 1; total protein was isolated for detecting expression of adiponectin and FABP4 by western blot. (b) Relative intensity levels were determined by densitometry of bands. Analyses were performed using ANOVA test. Data are expressed as mean ± SD and differences were considered significant with *P* < 0.05. ∗Differences between total and partial agonist in relation to control. # indicates meaningful differences observed after 4 hours of treatment with agonists after exposure to cold in comparison with adipocytes treated in equal conditions at 37°C. Data were normalized with GAPDH.

In comparison to effect of temperature over energy expenditure, the cold consequence on adipocytokines expression has been poorly studied. In the present study, we observed an increase in adiponectin expression in cells that were differentiated by both PPAR*γ* agonists (Figure 3(a)). This observation together with the decrease of the amount of triglycerides as well as an increase in the thermogenic markers of adipose cells directs us to believe that WAT cells from ADMSCs have browning process towards beige adipocytes. It is important to highlight the first occasion in which a reduction in temperature has a beneficial change in adiponectin production by adipose cells derived from ADMSCs, both at baseline and after therapy PPAR*γ* agonists (Figures 3(a) and 3(b)).

**Figure 4:**
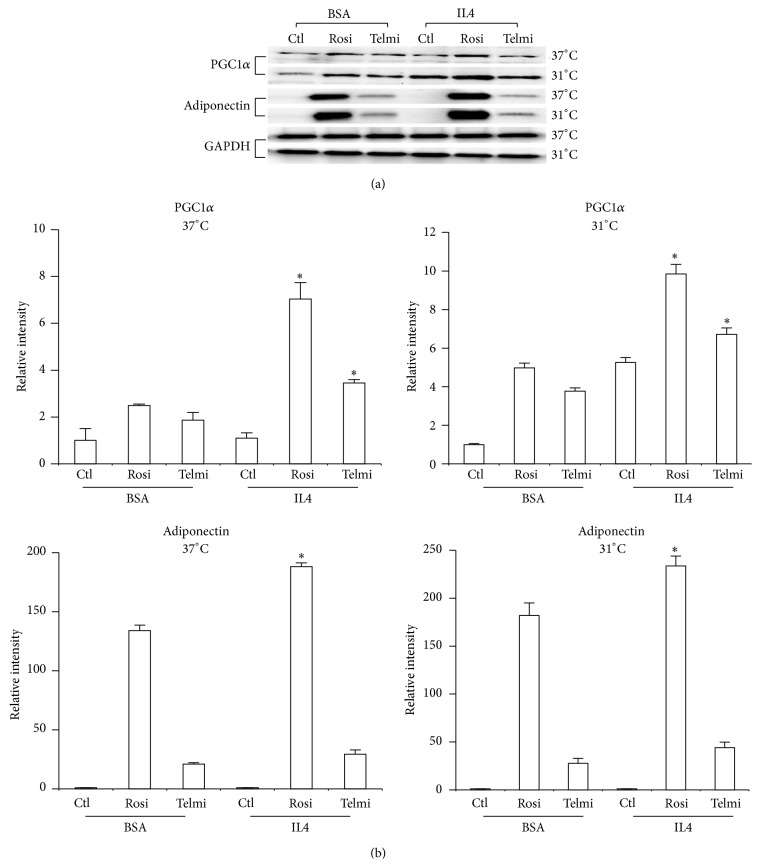
Effect of IL4 in the levels of adipocytokines. (a) The differentiated adipocytes were obtained and treated as describe before; total protein was isolated for detecting expression of adiponectin and PGC-1*α* by western blot. IL4 was added to cells during a period of 4 hours and at the same time cells were exposed to 37°C or 31°C. (b) Relative intensity levels were determined by densitometry of bands. Analyses were performed using ANOVA test. Data are expressed as mean ± SD and differences were considered significant with *P* < 0.05. ∗The differences between the cells treated with IL4.

Recently a new population of regulatory cells that mediate cold response was observed in mice alternatively activated (type 2/M2) macrophages. When activated by eosinophils via IL4 and IL13 signaling, M2 macrophages are recruited to subcutaneous WAT and secrete catecholamines to activate WAT browning [20, 29, 30]. We treated the differentiated adipose cells from ADMSCs with IL4 during a period of 4 hrs at 37°C and 31°C. IL4 increased the expression of adiponectin and PGC-1*α* and temperature reduction had synergistic effect with IL4 (Figures 4(a) and 4(b)). This leads us to make the hypothesis that IL4 is an important factor in the process of browning of scWAT from ADMSCs.

## 4. Conclusion

In the present study we demonstrate that partial activation of nuclear receptors PPAR*γ* may determine a change of ADMSCs lineage to adipocyte. Additionally, it was observed that both the reduction of temperature and the IL4 might have a protective metabolic effect from obesity.
